# Two Cases of Lymph Node Metastasis Found in Differentiated, Small-Sized Gastric Adenocarcinomas: Did Tumor Budding Play a Critical Role?

**DOI:** 10.3390/medicina59122126

**Published:** 2023-12-05

**Authors:** Young Sub Lee, Yosep Chong, Kyung Jin Seo, Kwangil Yim

**Affiliations:** 1 Department of Hospital Pathology, Eunpyeong St. Mary’s Hospital, College of Medicine, The Catholic University of Korea, Seoul 03312, Republic of Korea; lys@catholic.ac.kr; 2 Department of Hospital Pathology, Uijeongbu St. Mary’s Hospital, College of Medicine, The Catholic University of Korea, Seoul 06591, Republic of Korea; ychong@catholic.ac.kr (Y.C.); ywacko@catholic.ac.kr (K.J.S.)

**Keywords:** early gastric cancer, submucosal dissection, differentiated gastric adenocarcinoma, lymph node metastasis, tumor budding

## Abstract

*Background:* Endoscopic resection (ER) is a minimally invasive therapeutic approach for early gastric cancer (EGC), particularly for cases with a low risk of lymph node metastasis (LNM). Tumor budding (TB) has gained attention as a potential prognostic indicator for LNM in EGC. *Case Presentation:* We report two cases—a 73-year-old and an 81-year-old male patient—who presented with gastric adenocarcinoma. Both patients had small-sized, differentiated, and intramucosal adenocarcinomas. However, high-grade TBs per high-power field under ×200 magnification at the invasive front and LNMs were found in both cases. *Conclusions:* These cases conformed to the post-ER observation guidelines of the current treatment protocol, yet demonstrated LNMs. We found that TB could serve as an effective prognostic marker for LNM compared to traditional risk factors. The aim of this study is to re-examine the ability of TB to predict LNM in EGC, thereby providing an impetus for reconsideration and potential revision of the current treatment guidelines for EGC.

## 1. Introduction

Early gastric cancer (EGC) is an invasive carcinoma of the stomach involving only the mucosa or submucosa, regardless of lymph node status [1]. EGC is known to carry an excellent prognosis if the complete excision of the lesion is done with endoscopic resection (ER) or surgery at a lower risk of lymph node metastasis (LNM) [2]. EGC with risk factors such as deep submucosal invasion, poorly cohesive carcinoma components, and lymphovascular invasion can have a higher risk for LNM [3].

The Japanese Gastric Cancer Treatment Guidelines categorize the effectiveness of ER for gastric cancer into three levels: eCura A, B, or C, based on how completely the primary tumor is removed and the likelihood of LNM [1]. In cases classified as eCura A, patients are advised to undergo endoscopic examinations for annual monitoring [4]. For eCura B, more frequent surveillance is recommended, involving either annual or biannual endoscopy, along with abdominal ultrasonography or computed tomography (CT) scans to check for metastasis [5]. For cases falling under the category of eCura C, where there is a higher risk or evidence of LNM, gastrectomy with lymphadenectomy is advised as the standard treatment approach [6,7,8].

In addition, tumor budding (TB) is known as a poor prognostic marker and a risk factor for LNM in colorectal cancer and is defined as the presence of single or small clusters of less than five tumor cells at the invasive tumor front [9]. Recently, TB has emerged as a potential predictor of LNM in gastric cancer (GC) lesions [3,9,10,11,12,13,14,15,16].

Herein, we present rare cases of perigastric LNMs after curative ER or gastrectomy for EGC, despite their small size and well-differentiated histology. These occurrences could be anticipated by the presence of TB. Consequently, our objective is to investigate the viability of TB as an indicator of LNM in EGC cases.

## 2. Case Presentation

We conducted a comprehensive review of all patients with EGC who underwent surgery at two institutions regardless of previous ER from January 2010 to October 2023 (520 patients in total: Eunpyeong St. Mary’s Hospital, *n* = 168; Uijeongbu St. Mary’s Hospital, *n* = 352). Among these, 235 patients (Eunpyeong St. Mary’s Hospital, *n* = 95; Uijeongbu St. Mary’s Hospital, *n* = 140) met the criteria for observation after ER (eCura A or B) according to the Japanese Gastric Cancer Treatment Guidelines [1]. Notably, only two individuals, despite meeting the criteria, exhibited LNMs.

### 2.1. Case 1

**Figure 1:**
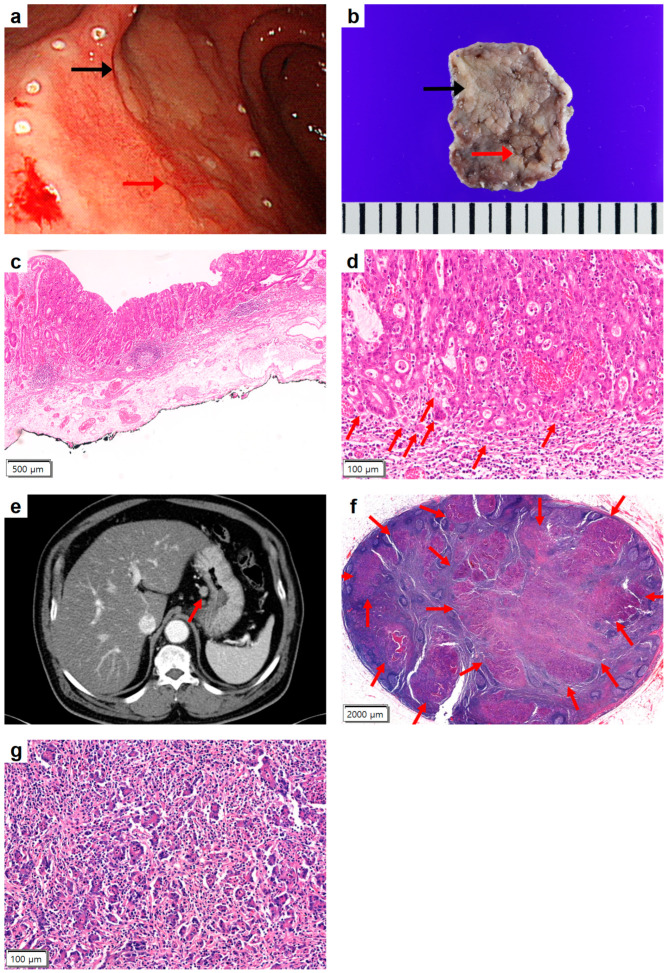
Endoscopic, radiologic, and pathologic findings of Case 1. (**a**) Esophagogastroduodenoscopy showed a flat lesion (red arrow) and a flat elevated lesion (black arrow) in the gastric antrum. The margin of the lesions had been demarcated using electrocautery in preparation for endoscopic submucosal dissection. (**b**) Gross examination of the endoscopic submucosal dissection specimen revealed a 2.0 cm × 2.0 cm flat lesion of early gastric cancer type IIb (red arrow) and a 3.0 cm × 3.0 cm flat elevated lesion of low-grade dysplasia (black arrow). (**c**) Low-power microscopic view of the early gastric cancer lesion showed moderately differentiated tubular adenocarcinoma with intact muscularis mucosa, which was confined to the lamina propria (hematoxylin–eosin [HE] stain, ×40). (**d**) High-power view of the invasive front of the early gastric cancer showed up to seven tumor buddings (arrows) (HE stain, ×200). (**e**) A follow-up abdominal computed tomography after a year revealed an enlarged lymph node (arrow) in the gastrohepatic ligament. (**f**) Histopathologic examination of the resected perigastric lymph node showed metastatic nests (arrows) in the lymph node (HE stain, ×12.5). (**g**) In high-power view, the nests were consistent with metastatic gastric tubular adenocarcinoma (HE stain, ×200).

A 73-year-old male presented to our outpatient clinic following the diagnosis of a moderately differentiated tubular adenocarcinoma, confirmed via an endoscopic biopsy. Upon endoscopic examination of the stomach, two distinct lesions were identified in the antrum: a 2.0 cm × 2.0 cm flat lesion showing characteristics indicative of a potential pathology, including an irregular surface, a discernibly reddish color, and poorly demarcated margins; and a 3.0 cm × 3.0 cm flat elevated lesion showing a paler, nodular surface with relatively well-defined boundaries (Figure 1a). Abdominal CT did not reveal any discernible gastric lesion, consistent with a clinical staging of cT1N0 [2].

Based on the clinicoradiological and endoscopic findings, an intramucosal and differentiated-type adenocarcinoma was diagnosed. The lesion was less than 3 cm in diameter, fulfilling the ‘Absolute Indication’ criteria for endoscopic submucosal dissection (ESD). Following ESD, the patient was discharged with no post-operative complications.

The pathological examination revealed two distinct lesions, as noted in the endoscopy (Figure 1b). The histologic findings of the flat lesion were consistent with moderately differentiated tubular adenocarcinoma, which was contained within the intramucosal layer, with intact muscularis mucosa (Figure 1c). The neoplastic mass measured was 0.7 cm × 0.7 cm, with clear resection margins. There was no evidence of lymphatic or venous invasion. Interestingly, up to seven foci of TB were noted per high-power field at the invasive front (Figure 1d). The histologic examination of the other flat elevated lesion revealed a low-grade dysplasia (2.7 cm × 2.0 cm) with clear resection margins.

The initial abdominal CT showed no evidence of abnormal lymphadenopathy. However, in the follow-up scan, a round lymphadenopathy 1 cm in size was observed in the gastrohepatic ligament (Figure 1e). Laparoscopic perigastric lymph node dissection was performed. Pathological examination of the resected lymph nodes confirmed that the lesion was a metastasis of the EGC (Figure 1f,g). A curative subtotal gastrectomy was performed two weeks later, with subsequent histopathological examination revealing no residual cancerous lesions in the primary site or lymph nodes. A follow-up was conducted five years post-surgery, and no signs of recurrence were observed.

### 2.2. Case 2

**Figure 2:**
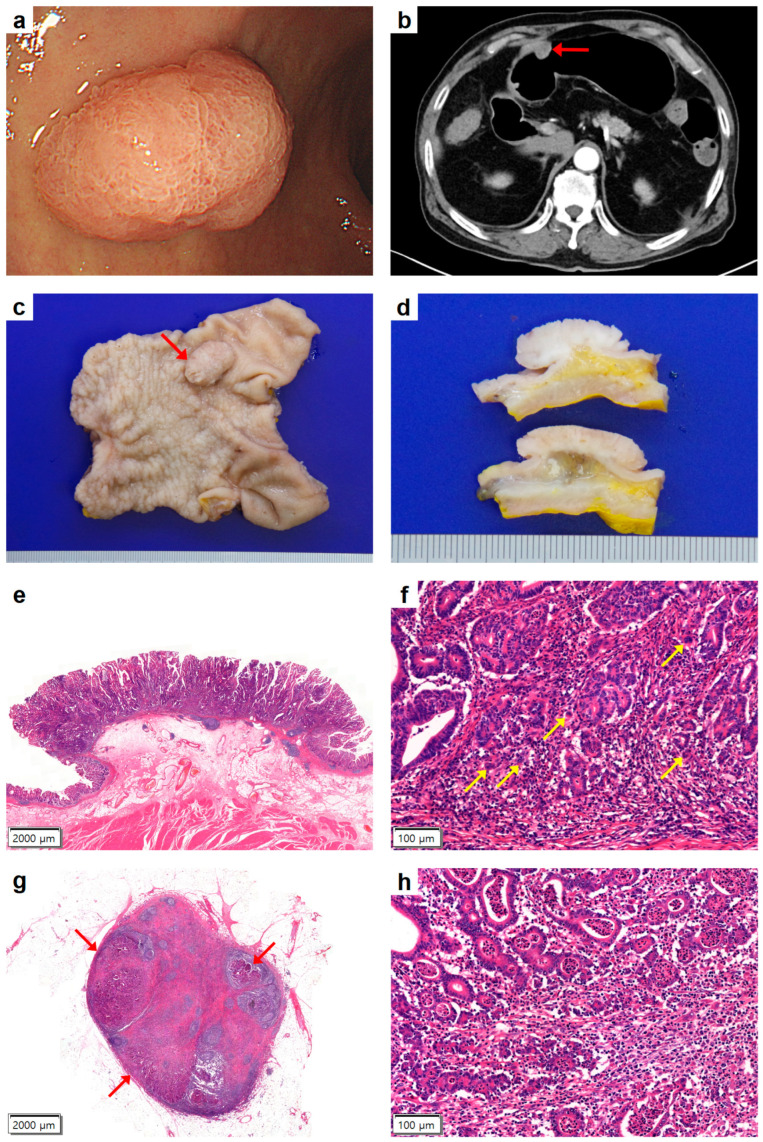
Endoscopic, radiologic, and pathologic findings of Case 2. (**a**) Esophagogastroduodenoscopy showed a polypoid mass without ulceration in the gastric antrum. (**b**) An initial abdominal computed tomography revealed a 2.0 cm sized protruding lesion (arrow) in the proximal antrum and no enlarged perigastric lymph node. (**c**) Gross examination of the distal subtotal gastrectomy specimen showed 2.3 cm × 1.5 cm sized elevated lesion of early gastric cancer type I (arrow) in the anterior wall of the antrum. (**d**) Serial sectioning revealed that the tumor was limited to the mucosa. (**e**) Low-power microscopic view of the tumor showed differentiated-type adenocarcinoma with intact muscularis mucosae (hematoxylin eosin [HE] stain, ×12.5). (**f**) High-power view revealed up to five foci of tumor buddings (arrows) at the invasive front (HE stain, ×200). (**g**) Although no lymphatic invasion was seen in the primary tumor, lymph node metastases (arrows) were found in perigastric lymph nodes (HE stain, ×12.5). (**h**) High-power view of the lymph nodes showed metastatic gastric adenocarcinoma (HE stain, ×200).

An 81-year-old male visited the outpatient clinic due to a gastric polyp found incidentally during a routine preventive health assessment. Endoscopic examination showed a polypoid lesion without an ulcer in the gastric antrum (Figure 2a). The endoscopic ultrasonographic diagnosis was EGC type I with suspected submucosal invasion. An endoscopic biopsy was performed, confirming gastric adenocarcinoma. The preoperative CT scan showed a 2.0 cm sized protruding lesion in the proximal antrum and no perigastric lymphadenopathy (Figure 2b). The patient underwent distal subtotal gastrectomy with D2 lymph node dissection because the possibility of advanced gastric cancer could not be completely ruled out.

However, gross examination revealed a 2.3 × 1.5-cm-sized protruding tumor without ulceration in the anterior wall of the proximal antrum (Figure 2c). On cut sections, the tumor was grossly confined to the mucosa (Figure 2d). Therefore, based on the clinicoradiological and gross findings, an intramucosal, differentiated-type adenocarcinoma without an ulcer was diagnosed.

Microscopic examination showed proliferation of irregularly shaped tubules of various sizes invading lamina propria only, which was consistent with intramucosal moderately differentiated tubular adenocarcinoma (Figure 2e). There was neither a lymphatic nor a vascular invasion. Furthermore, in the detailed analysis of the invasive front, up to five foci of TB were unveiled at the invasive front of the area of highest focus, as observed under one high-power field (Figure 2f). LNMs were identified in two lymph nodes in the perigastric area near the antrum and lesser curvature (Figure 2g,h). During the 4-year follow-up, no recurrence or distant metastasis was observed.

## 3. Discussion

The Japanese Gastric Cancer Association [1] delineates three distinct categories of GC lesions amenable to ER, based on the varying risk of LNM. The ‘Absolute Indication’ category pertains to tumors that qualify for ER as the standard treatment approach, having a less than 1% risk for LNM. On the other hand, the ‘Expanded Indication’ category is intended to include undifferentiated types of adenocarcinomas within the purview of ER. Although the LNM risk associated with this category might be less than 1%, evidence supporting this assertion remains unestablished [1]. Cases categorized as ‘Relative Indications,’ which fundamentally diverge from absolute or expanded indications, typically require a therapeutic approach. Nevertheless, ER maintains potential curative efficacy in these cases, particularly for individuals who are not fit for surgery [1].

Additionally, cases following ER were categorized into three groups: eCura A, B, and C. eCura A is defined by the following criteria: absence of lymphovascular invasion and intramucosal cancer (pT1a) with (1) no ulcer and differentiated type; (2) ulcer, differentiated type, and a tumor size less than 3 cm; or (3) no ulcer, undifferentiated type, and a tumor size less than 2 cm. eCura B is characterized by the absence of lymphovascular invasion, submucosal cancer (pT1b) with less than 500 µm invasion depth, differentiated type, and a tumor size less than 3 cm. Finally, eCura C includes all cases that do not fall into the categories of A or B. For eCura A, annual endoscopic surveillance is recommended [4]. In eCura B, more frequent surveillance is advised [5]. However, for eCura C, gastrectomy is considered the standard treatment [6,7,8]. In the present study, the GC lesions were confined to the intramucosal layer (pT1a) and measured less than 3 cm, with no lymphatic and venous invasion or margin involvement. Therefore, the lesions fell under the category of eCura A [1], making them eligible for observation.

Extragastric recurrence after ER was reported in 0.14–0.21% of cases [17,18,19], and three cases of LNM after curative ER for EGC have been reported in the literature [20,21,22]. Hanaoka et al. [21] reported a case of LNM and liver metastasis within 14 months after curative ER of a mixed moderately and poorly differentiated intramucosal EGC 5.5 cm in size. Fujii et al. [20] reported a case of LNM within 17 months after curative ER for moderately differentiated EGC 2.2 cm in size, which was confined to the mucosa with an ulcer. Also, Kamiya et al. [22] reported a case of LNM within four years after curative ER of a poorly differentiated EGC 3.0 cm in size. The association between TB and LNM has not been described in the previous reports.

In the present cases, despite the well-differentiation and small size of the primary tumors, several TBs were noted in the invasive front [3,9,10,11,12,13,14,15,16]. TB is associated with epithelial–mesenchymal transition [9,23] and is a well-known prognostic factor for colorectal cancer [9,24,25,26]. In various solid cancers, including biliary tract, breast, head and neck, lung, pancreas, and urinary tract cancers, TB has been recognized as a poor prognostic factor due to its close association with LNM [9,27,28,29,30,31,32,33,34,35]. TB has also emerged as a potential predictor of LNM in GC lesions [3,9,10,11,12,13,14,15,16].

In cases of EGC, the role of TB as a risk factor for LNM has been predominantly explored in intestinal-type GCs [9,13] or studied irrespective of the tumor’s pT stage [13,14,15]. Certain studies have focused exclusively on submucosal EGC [3] and employed the ‘presence/absence of TB (TB-YN)’ method alone [3,10]. For example, Du et al. [3] and Gulluoglu et al. [10] identified the presence of TB as an independent LNM risk factor in cohorts of 632 submucosal EGCs and 126 EGCs, respectively. Olsen et al. [13] correlated high-grade TB (median number of TB foci ≥1 in 10 × 200 fields) with LNM and a poor prognosis in a sample population of 16 EGCs. Tanaka et al. [14] associated high-grade TB (>10/×400 high-power fields) with LNM in a study of 65 EGCs. Ulase et al. [15] identified the presence of TB as a substantial risk factor for LNM in a cohort of 57 EGC cases. Lastly, Yim et al. [16], utilizing a quantitative approach, demonstrated that high-grade TB (defined as ≥5 TB foci in one representative slide) was associated with LNM.

## 4. Conclusions

We reported two cases that met eCura A criteria according to the current treatment guideline [1] but developed LNM. The present cases had many features indictive of a low potential for LNM, such as small tumor size, well differentiation, and intramucosal depth of invasion. A notable concern regarding LNM emerged from the observation that multiple foci of TB were present at the leading edge of the invasive front. Consequently, we suggest the possibility that TB may play a significant role in the development of LNM. Furthermore, we propose that describing the presence of TB in routine pathological reports could help predict the risk of recurrence after resection and decide on proper management in patients with EGC.

## Data Availability

The data presented in this study are available upon a reasonable request from the corresponding author.
